# Integrating Digital Health, Citizen Engagement, and Behavioral Science for Climate Change Mitigation

**DOI:** 10.1055/s-0046-1816616

**Published:** 2026-11-03

**Authors:** Pascal Staccini, Annie Y.S. Lau

**Affiliations:** 1 URE RETINES, Faculté de Médecine, Université Côte d'Azur, NiceFrance; 2 Center for Health Informatics, Australian Institute of Health Innovation, Macquarie UniversityAustralia

**Keywords:** Climate change, environmental health, digital health, citizen science, behavioral sciences, health literacy, social media, public health, mental health, health policy

## Abstract

**Objectives** : Identify how recent interdisciplinary research integrates digital health, citizen engagement, and behavioral science to address climate-related health challenges and inform mitigation strategies.

**Method** : A bibliographic search was conducted in PubMed using a structured query targeting the intersection of climate change, digital health and citizen participation, published between January 2024 and December 2024. An initial screening was performed based on titles and abstracts to assess relevance to the themes of climate-related health strategies. From this search, 15 articles were selected as “best paper candidates” through full-text review and expert consensus, based on their methodological quality, originality, and contribution to the field. Among these articles, two papers were ultimately designated as “best papers” following a peer-review process coordinated within the Yearbook of Medical Informatics selection framework.

**Results** : Among the 174 returned papers published in 2024 across the various areas of the Consumer Health Informatics section, the full review process selected two best papers and identified 13 additional articles as strong candidates contributing to the intersection of climate change and health. The thematic synthesis of these 15 articles revealed eight core themes, organized under three strategic pillars: digital health, citizen engagement, and behavioral science. Within the digital health pillar, studies explored the role of mobile technologies, wearable sensors, and digital platforms in monitoring environmental exposures and informing adaptive health behaviors, while also examining the environmental footprint of digital infrastructures themselves. Articles grouped under citizen engagement emphasized the rise of participatory research models, such as citizen science and community-based environmental monitoring, alongside the urgent need to reform environmental health education among future health professionals. Contributions to the behavioral science pillar included large-scale experimental studies evaluating the effectiveness of nudges and framing strategies on climate-relevant beliefs and behaviors, as well as investigations into the mental health impacts of climate-related stressors and the protective role of social support and nature connectedness. Collectively, these findings reflect a dynamic and interdisciplinary research landscape, pointing to the growing integration of technological, civic, and psychological approaches in health-centered climate change mitigation strategies.

**Conclusions** : This review highlights how recent interdisciplinary research integrates digital health, citizen engagement, and behavioral science to address the health impacts of climate change. The selected studies demonstrate a growing convergence of technological innovation, participatory methods, and behavioral insights in climate-health strategies. While digital tools enhance exposure monitoring and risk communication, they also raise concerns about sustainability and equity. Citizen-driven initiatives and behavioral interventions emerge as essential but context-sensitive components of adaptive responses. Together, these findings support the need for integrated, inclusive, and evidence-based frameworks to guide health system transformation in the face of ecological crises.

## 1. Introduction

The health consequences of climate change are increasingly recognized as urgent, complex, and multifactorial, demanding coordinated action across many disciplines, sectors, and governance levels. Rising temperatures, pollution, and ecological disruptions contribute to a growing burden of disease and deepen existing health inequities on a global scale. In response, researchers, policymakers, and civil society actors are exploring new frameworks that integrate technological innovation, public engagement, and behaviorally informed strategies [ 1 ].

Digital health technologies—such as mobile apps, wearables, and integrated data platforms—facilitate the monitoring of environmental exposures, the delivery of tailored health information, and the encouragement of adaptive behaviors. However, their development and deployment raise critical questions concerning user acceptance, equitable access, long-term usability, and environmental sustainability [ 2 ].

In parallel, citizen engagement has become a central component of health and environmental research. Initiatives such as citizen science, participatory policy-making, and community-based surveillance challenge conventional hierarchies of knowledge and create new possibilities for inclusive, data-driven governance. Yet, they also generate tensions around data reliability, ethical participation, and measurable impact [ 3 ].

Behavioral science provides robust conceptual and empirical frameworks to understand the psychological drivers of environmental and health-related decisions. Nevertheless, its implementation in climate-health contexts remains heterogeneous, often shaped by local cultural, political, and structural factors.

Taken together, these evolving dynamics prompt several critical questions: To what extent are digital technologies effectively used to mitigate climate-related health impacts, and how do the technologies account for their own ecological footprint? Is citizen participation being conceptualized merely as a tool for data collection, or as a deeper process of co-construction? What types of behavioral strategies succeed in shifting climate-relevant health behaviors—and for which populations? Finally, is mental health, including climate-related anxiety and resilience, adequately integrated as a dimension of climate adaptation?

These questions define a fast-moving field of inquiry and highlight the need for interdisciplinary approaches that are integrative, responsive, and ethically grounded in the context of ecological disruption.

## 2. About the paper Selection

We conducted a structured literature search using PubMed to identify articles published in 2024. The aim of this analysis was to provide a perspective on the intersection between climate change and Consumer Health Informatics (CHI), with particular emphasis on citizen science—a methodological lens we applied in our CHI review the previous year. While our previous methodology relied on the PICO framework with clearly defined components, this year's analysis adopted a more focused and integrative strategy. Specifically, we concentrated on three thematic pillars: digital health, social media, and climate change. The PubMed query was constructed accordingly and is outlined in the four sections below:

**Table:** 

i.
(2024/1/1:2024/12/31[DP] NOT “Epub ahead of print”) NOT Bibliography[pt] NOT Comment[pt] NOT Editorial[pt] NOT Letter[pt] NOT News[pt] NOT Case Reports[pt] NOT Published Erratum[pt] NOT Historical Article[pt] NOT Legal Case[pt] NOT legislation[pt] NOT (systematic review[pt] OR “review literature as topic”[MeSH Terms] OR “review”[All Fields])

**Table:** 

ii.
AND (“digital health”[All Fields] OR “ehealth”[All Fields] OR “e-health”[All Fields] OR “tele-health”[All Fields] OR “mhealth”[All Fields] OR “m-health”[All Fields] OR “mobile health”[All Fields] OR “telemedicine”[All Fields] OR “electronic patient-physician communication”[All Fields] OR “electronic medical record”[All Fields] “electronic health record”[All Fields] OR “personal health record”[All Fields] OR “electronic patient record”[All Fields] OR “electronic”[All Fields] OR “information technology”[All Fields] OR “communication technology”[All Fields] OR “mobile”[All Fields] OR “cell phone”[All Fields] OR “smart phone”[All Fields] OR “online”[All Fields] OR “online systems”[All Fields] OR “internet”[All Fields] OR “web”[All Fields] OR “website”[All Fields] OR “patient portal”[All Fields] OR “tele*”[All Fields] OR “digital*”[All Fields] OR “remote*”[All Fields] OR “video*”[All Fields] OR “virtual”[All Fields] OR “virtuality”[All Fields] OR “virtualization”[All Fields] OR “virtualized”[All Fields] OR “virtualizing”[All Fields] OR “virtuals”[All Fields])

**Table:** 

iii.
AND (“social media”[All Fields] OR “facebook”[All Fields] OR “twitter”[All Fields] OR “youtube”[All Fields] OR “instagram”[All Fields] OR “social network site”[All Fields] OR “social web”[All Fields] OR “online social network”[All Fields] OR “social environment”[All Fields] OR “social process”[All Fields] OR “social competition”[All Fields] OR “social norm”[All Fields] OR “social feedback”[All Fields] OR “social influence”[All Fields] OR “social comparison”[All Fields] OR “social network”[All Fields] OR “discussion group”[All Fields] OR “support group”[All Fields] OR “social support”[All Fields] OR “community network”[All Fields] OR “online community”[All Fields] OR “Patient Participation”[ All Fields] OR “Citizen Science”[All Fields] OR “Community Health Services”[ All Fields])

**Table:** 

iv.
AND (“Climate Change”[ All Fields] OR “Global Warming”[ All Fields] OR “Environmental Health”[ All Fields] OR “Air Pollution”[ All Fields] OR “Greenhouse Effect”[ All Fields] OR “Environmental Exposure”[ All Fields] OR “Ecosystem”[ All Fields] OR “Sea level rise”[ All Fields] OR “Climatic processes”[ All Fields])

A total of 174 articles were retrieved through the PubMed search. From this corpus, fifteen candidate papers were identified after independent evaluation by co-editors using the Rayyan web tool [ 4 ], followed by a consensus discussion. Screening decisions were based on four main criteria: i) relevance to the 2024 Yearbook theme “Digital Health and Climate Change”; ii) a focus on patients and consumers; iii) the scope and nature of the issues addressed; and iv4) the novelty of the methodological approach. The shortlisted articles were then reviewed and scored by an international panel of experts, following the IMIA Yearbook's best paper selection protocol. Final decisions were made during the annual IMIA Yearbook board meeting.

This synopsis highlights the two papers ultimately selected as best contributions. Froeling *et al.* [ 5 ] present a compelling case study in co-produced environmental epidemiology, illustrating how citizen engagement can enhance data quality, community trust, and policy relevance in exposure assessment. Their work exemplifies a broader shift toward democratized and inclusive research practices, with significant implications for public health governance.

In contrast, Vlasceanu *et al.* [ 6 ] report one of the largest international intervention studies in behavioral climate science. They tested eleven behavioral strategies across 63 countries, involving more than 59,000 participants. Their findings offer nuanced insight into the heterogeneous effectiveness of behavioral nudges in shaping climate beliefs, policy support, and engagement. The study reveals clear variations influenced by cultural and psychological factors.

Together, these two studies not only advance academic understanding but also demonstrate innovative, scalable models for addressing climate-related health challenges.

## 3. Outlook

Climate change poses an unprecedented threat to human health. It worsens both chronic and infectious diseases, disrupts health systems, and intensifies the social and environmental determinants of vulnerability. Mitigating climate change health impacts requires more than ecological or technological change—it calls for approaches that are human-centered, systems-oriented, and grounded in evidence.

In this context, the convergence of digital health, citizen engagement, and behavioral science provides a compelling interdisciplinary framework to address the health dimensions of the climate crisis. This integrated approach draws on emerging technologies, participatory research models, and psychological insights to anticipate climate-related risks and foster adaptive responses. These responses span multiple levels of action—from individual behaviors to community resilience and institutional transformation.

### 3.1 Digital Health

The first pillar, Digital Health, refers to the use of information and communication technologies to monitor, assess, and respond to environmental health risks in real time. This includes the development of climate-responsive digital infrastructures—such as mobile health applications, wearable sensors, and social media analytics—designed to track air pollution exposure, behavioral adaptation, and public sentiment. Importantly, digital health also interrogates its own environmental footprint, confronting the paradox that technologies intended to mitigate climate harms may themselves contribute to ecological degradation. Beyond personal health monitoring, digital health systems are increasingly integrated into the built environment. For example, urban exposure analytics now combine spatial data, pollution modeling, and social health indicators to inform the design of more resilient, adaptive cities.

#### 3.1.1 Digital Health for Environmental and Climate-Responsive Healthcare

Digital health technologies are becoming central to climate-related health surveillance, communication, and adaptation. Yet, as Istrate *et al.* [ 7 ] show, digital activities like streaming and video conferencing can account for up to 40% of an individual's carbon budget under the 1.5°C climate target—highlighting their dual role as both solutions and contributors to environmental harm. To mitigate risks while enhancing responsiveness, Katapally and Bhawra [ 8 ] advocate for digital dashboards powered by citizen-generated data, enabling real-time, cross-sectoral support for vulnerable populations. Yang *et al.* [ 9 ] reveal how individuals in 83 cities adjust physical activity in response to air quality alerts from wearable sensors, with behavior shaped by information trust, including peer and social media sources. Similarly, Lane *et al.* [ 10 ] find that climate-related tweet patterns inversely correlate with obesity rates, suggesting that digital discourse may mirror or modulate environmental health awareness. Collectively, these studies affirm that responsibly designed and sustainably powered digital health infrastructures can support anticipatory action, behavioral adaptation, and equitable responses to climate-related health stressors.

#### 3.1.2 Environmental Sustainability of Digital Infrastructures

Istrate *et al.* [ 7 ] conducted a life cycle assessment of digital media use, revealing that activities like video streaming and conferencing significantly contribute to environmental impacts—including carbon emissions, mineral depletion, and freshwater toxicity. They estimate that digital content consumption alone may account for up to 55% of the per capita limit for mineral and metal resource extraction. While decarbonizing power grids may reduce emissions, material resource strain remains unresolved, challenging the assumption that digital transformation is inherently sustainable. The authors call for extending device lifespans, minimizing redundancy, and integrating environmental criteria into digital health policies. Crucially, they argue that environmental costs—encompassing carbon, supply chain ethics, and e-waste—must be central to evaluating digital health's net benefit in climate mitigation.

#### 3.1.3 Urban Environmental Exposures and Social Health

Zhang *et al.* [ 11 ] examined how residential greenness and air pollution interact to influence social health in dense urban settings like Hong Kong. Using survey data from 1,977 adults and deep-learning-derived environmental metrics, they found that street-view greenness and park density correlated positively with social contact and relational support, whereas higher levels of O _3_ and NO _2_ were linked to reduced social cohesion. Through Partial Least Squares Structural Equation Modelling, they showed that pollution harms social health directly and indirectly via emotional distress, with physical activity and negative emotions acting as mediators. Effects varied by age, socioeconomic status, and employment, underscoring environmental inequities in access to urban green space. The findings highlight the role of urban design—especially green infrastructure and air quality improvements—in enhancing community resilience and mental well-being, and support treating such interventions as complementary to digital health when informed by sensor and spatial data.

### 3.2 Citizen Engagement

The second pillar, Citizen Engagement, emphasizes the active involvement of non-specialists in co-producing and sharing climate-health knowledge. Once peripheral, citizen science and participatory epidemiology are now central to enhancing trust, inclusivity, and data richness in environmental health. At the same time, the rising need for climate-literate healthcare professionals exposes curricular gaps in medical and public health education. Meanwhile, digital engagement—especially among youth and marginalized communities via social media—is transforming climate advocacy, prompting new approaches to public health communication and digital governance.

#### 3.2.1 Citizen Science and Participatory Research in Climate-Related Environmental Health

Citizen science in environmental health has progressed from isolated monitoring efforts to structured, co-designed research frameworks. Laurent *et al.* [ 12 ] developed the LILAS initiative, using an analytical matrix to guide civil society's engagement in studies of exposure, risk, and epidemiology—highlighting benefits such as data relevance, legitimacy, and empowerment. Froeling *et al.* [ 5 ] applied this model in five European studies through the CitieS-Health project, involving citizens in all research phases—from question design to dissemination—on topics ranging from industrial pollution to air quality and mental health. While underscoring the value of co-creation, they also noted challenges related to time, scientific rigor, and participant expectations. Extending this approach, Bousiotis *et al.* [ 13 ] used mobile citizen-sensing in Birmingham to detect hyperlocal PM _10_ and black carbon levels linked to construction and traffic, revealing patterns missed by conventional monitors. Together, these studies establish citizen science as a core methodology for socially responsive and fine-grained environmental health research.

#### 3.2.2 Educational Gaps and Curriculum Reform in Environmental Health

Grobon *et al.* [ 14 ] surveyed French medical students to assess their exposure to and demand for environmental health education. They found that 62.7% had received no formal training, and 63.6% reported no knowledge in the area—relying instead on non-peer-reviewed digital sources, including social media, raising concerns about misinformation. Despite this gap, 87.3% expressed interest in a dedicated course, highlighting strong curricular demand. The authors argue for the structured integration of environmental health into medical education—covering air pollution, toxicology, planetary health, and climate mitigation—while also promoting digital health literacy to enable critical appraisal of online information. The study positions education reform as essential for empowering future clinicians and strengthening health systems' resilience to climate change.

#### 3.2.3 Digital Engagement for Youth and Climate Awareness

Young people increasingly use digital platforms not just for information but as tools for climate mobilization. Arnot *et al.* [ 15 ] found that Australian youth view platforms like TikTok as inclusive, emotionally powerful spaces for climate advocacy, though concerns about misinformation and limited policy impact persist. Complementing this, Carneiro and Tucci [ 16 ] analyzed a decade of Twitter data, showing through text mining and sentiment analysis that climate discourse increasingly links environmental, health, and security concerns. Lane *et al.* [ 10 ] added a public health perspective, finding that counties with more environmentally focused tweets tend to have lower obesity rates, even after controlling for socioeconomic factors—suggesting a link between digital discourse and local health awareness. Collectively, these studies frame digital engagement as active participatory citizenship, with youth emerging as both vulnerable populations and vital agents in climate-health action.

### 3.3 Behavioral Science

The third pillar, Behavioral Science, offers critical insights into how individual and collective behaviors shape responses to climate change. Large-scale international studies have shown that behavioral interventions vary in their effectiveness, underscoring the need for context-sensitive, psychologically grounded strategies. At the same time, rising levels of eco-anxiety and emotional disengagement highlight the urgency of integrating mental health and resilience into climate adaptation. Interventions fostering nature connectedness, social support, and mindful information use are now recognized as essential components of climate-resilient health systems.

#### 3.3.1. Behavioral Science Approaches to Climate Action

Behavioral science provides evidence-based tools for fostering climate action through individual and collective behavior change. Doell *et al.* [ 17 ] surveyed over 59,000 people across 63 countries to identify psychological and sociodemographic drivers of climate attitudes, policy support, and environmental behaviors. Their findings informed cross-cultural strategies for engagement. Expanding on this, Vlasceanu *et al.* [ 6 ] tested 11 behavioral nudges in the same global cohort, revealing that intervention effects varied by outcome and audience. While psychological distance and empathy nudges modestly increased belief and policy support, no intervention significantly boosted real-world actions like tree planting—some even decreased it. Emotional appeals proved more effective than rational arguments in driving information sharing. The results highlight that behavioral interventions must be tailored to context, audience, and desired outcomes, particularly given reduced efficacy among climate skeptics whose resistance is rooted in identity rather than knowledge gaps. Together, these studies emphasize the importance of psychologically nuanced, culturally adaptable strategies in climate-health behavior change.

#### 3.3.2 Mental Health and Psychological Resilience Amid Ecological Crises

The psychological impacts of climate change are increasingly evident, particularly among youth and vulnerable groups. Lau *et al.* [ 18 ] surveyed 2,000 young adults across five countries and found that emotional engagement with global crises—especially ecological ones—predicted poorer mental health, unless moderated by strong nature connectedness and mindful media use. Structural equation modelling showed that nature exposure reduced stress and depression, while social media had both protective and harmful effects depending on content and frequency. Complementing this, Hassani *et al.* [ 19 ] found that emotional and instrumental support significantly lowered stress in diabetic patients during the COVID-19 pandemic, reinforcing social cohesion as a key resilience factor—especially relevant amid rising climate-related infectious threats. Additionally, Doell *et al.* [ 17 ] incorporated mental health indicators into their global survey, offering a broad psychosocial profile of climate concern. Collectively, these studies argue for embedding mental health services into climate adaptation, promoting “psychological resilience infrastructures” that integrate nature, social support, and responsible media engagement.

## 4. Conclusion

This review of fifteen recent peer-reviewed articles highlights a growing convergence between digital health infrastructures, civic participation, and behavioral science as foundational levers for mitigating the health consequences of climate change. Digital health emerges both as a facilitator and a paradox: while mobile technologies, wearables, and digital communication platforms enhance real-time environmental surveillance and enable adaptive behavioral responses, they simultaneously contribute to energy-intensive consumption patterns that risk exceeding planetary boundaries. Studies addressing urban greenness, air pollution, and the environmental footprint of digital infrastructures underscore the urgent need for a digital health paradigm that is not only technologically advanced but also ecologically sustainable.

Concurrently, the role of citizen engagement is evolving from passive data contribution to active co-production of scientific knowledge, public health interventions, and policy discourse. Citizen science initiatives detailed in this corpus demonstrate both the methodological robustness and the ethical imperative of involving lay populations in climate-health research. These participatory approaches enhance data granularity, contextual relevance, and public trust. However, findings also reveal structural deficits in health education—particularly within medical training—where environmental health remains underrepresented despite its significant share of the global disease burden. This educational lag constitutes a barrier to systemic readiness and must be addressed through curricular reforms that foreground planetary health competencies.

Digital platforms are also shown to function as influential arenas for political mobilization and emotional expression, especially among younger populations. Social media channels not only amplify climate advocacy but also provide psychosocial outlets that mediate affective responses to ecological threats. These digital expressions, far from being merely symbolic, correlate with tangible public health indicators and illuminate broader dynamics of risk perception, identity, and trust in information systems.

Articles grounded in behavioral science further demonstrate that climate-related attitudes and behaviors are indeed modifiable, yet not uniformly across populations. While nudges and framing interventions can shift beliefs and intentions, their impact diminishes among climate skeptics and in contexts requiring high-effort behavioral change. The evidence supports the design of tailored, culturally sensitive interventions that integrate psychological insights—such as motivational dynamics and perceived psychological distance—to optimize behavioral outcomes.

Crucially, several studies draw attention to the mental health burden imposed by ecological instability. Rising levels of climate anxiety and emotional exhaustion, particularly among youth and at-risk populations, call for integrative resilience strategies. These include enhancing social support, promoting nature connectedness, and fostering critical media literacy to buffer the psychological impacts of climate-related uncertainty.

Taken together, the reviewed articles illustrate the indispensability of a transdisciplinary approach. Technological innovation, civic engagement, and behavioral insight must be strategically aligned—not treated as siloed domains—to build climate-resilient health systems. This integrated framework not only facilitates mitigation and adaptation but also expands the anticipatory and participatory capacity of health governance. It shifts the climate-health agenda away from technocratic solutionism toward a model of inclusive, equity-oriented innovation. In sum, these fifteen studies collectively chart a multidimensional, empirically grounded roadmap for transforming how health systems conceptualize and respond to the challenges posed by a changing climate.

**Table 1.** Selection of best papers for the 2024 IMIA Yearbook of Medical Informatics for the section Consumer Health Informatics. The articles are listed in alphabetical order of the first author's surname.

Section Education, Consumer Health Informatics, and Citizen Science
• Froeling F, Gignac F, Toran R, Ortiz R, Ficorilli A, De Marchi B et al. Implementing co-created citizen science in five environmental epidemiological studies in the CitieS-Health project. Environ Res. 2024 Jan 1;240(Pt 2):117469. https://doi.org/10.1016/j.envres.2023.117469 . Epub 2023 Oct 21. PMID: 37871787. • Vlasceanu M, Doell KC, Bak-Coleman JB, Todorova B, Berkebile-Weinberg MM, Grayson SJ et al. Addressing climate change with behavioral science: A global intervention tournament in 63 countries. Sci Adv. 2024 Feb 9;10(6):eadj5778. https://doi.org/10.1126/sciadv.adj5778 . Epub 2024 Feb 7. PMID: 38324680; PMCID: PMC10849597.
